# COVID-19 Mask Usage and Social Distancing in Social Media Images: Large-scale Deep Learning Analysis

**DOI:** 10.2196/26868

**Published:** 2022-01-18

**Authors:** Asmit Kumar Singh, Paras Mehan, Divyanshu Sharma, Rohan Pandey, Tavpritesh Sethi, Ponnurangam Kumaraguru

**Affiliations:** 1 Indraprastha Institute of Information Technology Delhi New Delhi India; 2 Netaji Subhas University of Technology New Delhi India; 3 Shiv Nadar University Greater Noida India

**Keywords:** COVID-19, mask detection, deep learning, classification, segmentation, social media analysis

## Abstract

**Background:**

The adoption of nonpharmaceutical interventions and their surveillance are critical for detecting and stopping possible transmission routes of COVID-19. A study of the effects of these interventions can help shape public health decisions. The efficacy of nonpharmaceutical interventions can be affected by public behaviors in events, such as protests. We examined mask use and mask fit in the United States, from social media images, especially during the Black Lives Matter (BLM) protests, representing the first large-scale public gatherings in the pandemic.

**Objective:**

This study assessed the use and fit of face masks and social distancing in the United States and events of large physical gatherings through public social media images from 6 cities and BLM protests.

**Methods:**

We collected and analyzed 2.04 million public social media images from New York City, Dallas, Seattle, New Orleans, Boston, and Minneapolis between February 1, 2020, and May 31, 2020. We evaluated correlations between online mask usage trends and COVID-19 cases. We looked for significant changes in mask use patterns and group posting around important policy decisions. For BLM protests, we analyzed 195,452 posts from New York and Minneapolis from May 25, 2020, to July 15, 2020. We looked at differences in adopting the preventive measures in the BLM protests through the mask fit score.

**Results:**

The average percentage of group pictures dropped from 8.05% to 4.65% after the lockdown week. New York City, Dallas, Seattle, New Orleans, Boston, and Minneapolis observed increases of 5.0%, 7.4%, 7.4%, 6.5%, 5.6%, and 7.1%, respectively, in mask use between February 2020 and May 2020. Boston and Minneapolis observed significant increases of 3.0% and 7.4%, respectively, in mask use after the mask mandates. Differences of 6.2% and 8.3% were found in group pictures between BLM posts and non-BLM posts for New York City and Minneapolis, respectively. In contrast, the differences in the percentage of masked faces in group pictures between BLM and non-BLM posts were 29.0% and 20.1% for New York City and Minneapolis, respectively. Across protests, 35% of individuals wore a mask with a fit score greater than 80%.

**Conclusions:**

The study found a significant drop in group posting when the stay-at-home laws were applied and a significant increase in mask use for 2 of 3 cities where masks were mandated. Although a positive trend toward mask use and social distancing was observed, a high percentage of posts showed disregard for the guidelines. BLM-related posts captured the lack of seriousness to safety measures, with a high percentage of group pictures and low mask fit scores. Thus, the methodology provides a directional indication of how government policies can be indirectly monitored through social media.

## Introduction

The outbreak of COVID-19 has the world in its grips. The World Health Organization declared it as a global pandemic on March 11, 2020 [1], and with exponentially rising cases, there are currently more than 32 million cases and 500,000 deaths in the United States (April 2021) [2]. Following World Health Organization health advisories, most countries have declared national emergencies, closed borders, and restricted public movement [3,4]. Masks have been found to reduce potential exposure risk from an infected person, proving to be a successful measure to suppress transmission and save lives [5-8]. Many studies have recognized the importance of community-wide use of masks for controlling the pandemic [9,10]. Social distancing measures have also been applied to prevent sick individuals from coming into contact with healthy individuals. These social distancing and mask use measures have proven successful in many countries like China [11,12]. Governments worldwide have adopted social distancing and mask use as primary nonpharmaceutical measures against the virus.

With over 32 million cases and 500,000 deaths as of April 2021, the United States is one of the largest countries to be hit by the virus. In the United States, many state governments had applied several stay-at-home and mask use measures as early nonpharmaceutical interventions. In the lead up to widespread vaccine deployment, the adoption of nonpharmaceutical interventions and their surveillance are critical for detecting and stopping possible transmission routes. Quantifying the effectiveness of such measures is a challenging task, which currently relies on on-ground surveys [13] or self-reported numbers [7]. However, these methods are cumbersome, thus leading to lags in data and the day-to-day evolution of a fast-moving pandemic.

The pervasive nature of social media provides a unique opportunity to create agile frameworks for assessing public health measures such as mask use. With its ease of access and global outreach, social media has a disproportionate influence on the dissemination of information during a pandemic [14]. In recent times of the pandemic, social media has become a popular platform for people to express their thoughts and opinions, and broadcast activities. The general public and authorities have been using hashtags like #CoronaOutbreak, #COVID19, and #mask to disseminate important information and health advisories, and this provides us with an opportunity to analyze behaviors and the impact of such advisories worldwide [15]. Indeed, social media has been extensively explored and analyzed for patterns that have emerged during the COVID-19 pandemic [16-19]. Signorini et al [20] examined Twitter based-information to track the swiftly evolving public sentiment regarding Swine Flu in 2011 and correlate the H1N1 virus subtype–related activity to track reported disease levels in the United States accurately.

During the pandemic, the United States also observed the Black Lives Matter (BLM) protests. The killing of George Floyd on May 25, 2020, sparked a series of protests [21] and agitations across the country. Protests are designed to stimulate public action for social justice. Such protests involve the physical gathering of people, making it difficult to adhere to social distancing and to wear masks. These protests provide an opportunity to observe how people react to public health–related preventive measures during such gatherings, but collecting the necessary data from the ground is a difficult task. Such protests have also gained high popularity and attraction through online social media [22,23].

Realizing the potential of social media in understanding such events, in this study, we used social media images from Instagram, a popular image-sharing social media platform, which has been used by researchers to study different public health emergencies [24]. Computer vision–based classifiers are necessary to check if a person is wearing a mask from an image. There exist 2 data sets previously published for mask classification tasks, namely, MAFA (Masked Faces) [25] and RMFD (Real-world Masked Face Dataset) [26]. The MAFA data set contains 35,805 masked images. Since the MAFA data set was curated and released in 2017, it could not capture different varieties and types of masks that have been in use during the pandemic period. The MAFA data set is biased toward 1 kind of mask; it majorly consists of medical staff wearing disposable medical-grade masks. The RMFD contains 7959 masked images with a variety of masks used during the COVID-19 period. However, a manual qualitative evaluation of the images revealed that the images were not suitable for analyzing high-quality social media images since most images were less than 50×50 resolution after cropping the face region. In addition to mask detection, analyzing the fit of the mask is a highly useful application. There is no previous work trying to analyze mask fit using semantic segmentation to the best of our knowledge.

Therefore, this study fills the gap with a pipeline designed to estimate the extent of mask behaviors by assessing mask use and mask fit from 2.04 million social media images obtained from 6 US cities. Along with geographical diversity among the cities, the 6 cities also have high population numbers. These cities were also found to have a high number of location-tagged posts on Instagram and hence were chosen as the locations of interest. We demonstrate the correlation of mask use and mask fit behaviors with COVID-19 burden, policy directives, and large-scale events, such as the nationwide BLM protests, in these 6 cities.

## Methods

### Data Sets

The study was approved by the institutional review board for adherence to ethical principles of research. The images were anonymized, and aggregated statistics for states were calculated. There was no attempt to recruit subjects, reidentify subjects, or link the images with other personal information in order to maintain confidentiality. Individual-level mask use adherence was not analyzed. Anonymized images were stored on secure servers as the following 3 different data set collections:

1. Mask-unmask classifier data set: This data set was used for training a model that classifies whether the person in the image is wearing a mask or not. For training, we needed images in which people were wearing masks (masked images) and images in which people were not wearing masks (unmasked images) so that our model could learn to distinguish between the 2 categories. We collected around 30,000 images of people wearing masks from Google Search images using the tags “people wearing masks” and “children wearing masks.” Images from Instagram were also collected with the tag explore feature, using the following 3 tags: “mask,” “masked,” and “covidmask.” Although the tagging algorithms used by Google are expected to capture most of the images, some images may have been missed. There is further scope for expanding our set of tags chosen to capture the entire population of images in which people are wearing masks, which is a limitation of our current approach. However, this will need more research, as capturing other scenarios may also lead to noisier sets. After data collection, images with a width and height of at least 50 pixels were kept to ensure decent image quality. Then, Face Detector was used on these images to extract faces. The images of extracted faces were distributed among 5 annotators, and the annotators were asked to classify the faces as either “masked” or “unmasked.” After the annotations, images of 9055 masked faces were obtained. For the unmasked face images, we created a random sample (without replacement) of 9055 faces from the VGGFace2 data set [27], which is a large-scale face recognition data set. The samples from VGGFace2 and the mask-unmask classifier data set were used to train the unmask-mask classifier, whose details are given in the Proposed Framework section.

2. Fit score data set: Out of 9055 masked faces that we obtained from the previous data set, we selected 504 images with different poses and a wide variety of mask designs. Then, we annotated these images using Label Studio [28] for getting pixel-level annotations of the mask region on the face. This data set was then used to train a semantic segmentation model, whose details are in the Proposed Framework section.

3. USA cities Instagram data set: For the analysis phase, we collected location-tagged public posts from Instagram between February 1, 2020, and May 31, 2020, for the following 6 cities: New York City, Seattle, Dallas, New Orleans, Minneapolis, and Boston. The first COVID case was reported in January 2020 [29], and till July 2020, the United States was still in the first wave of the COVID-19 pandemic [30]. Hence, the chosen time frame captures the beginning and growth of the COVID-19 pandemic in the United States. This collection was done for 6 major US cities, and these 6 cities were selected to represent different geographical sections of the country. We collected a total of 2.04 million public posts from these 6 cities. These posts were collected via Instagram’s explore location feature. Instagram’s GraphQL application programming interface was employed for the data collection. The tools used have been published as a python PyPI package [31]. We also collected 195,452 posts for New York City and Minneapolis from May 25, 2020, to July 15, 2020, which had major protests [32]. We curated a list of trending tags and keywords (“blm,” “blacklivesmatter,” “georgefloyd,” “justiceforgeorgefloyd,” “policebrutality,” and “protest”) during this period. We refer to the posts whose captions included these tags as BLM posts and the rest as non-BLM posts.

### Proposed Framework

This article proposes a mask-unmask classification framework (for classifying masked and unmasked images) and a fit score analysis framework (for evaluating whether the masks are being worn effectively or not in the given image). The mask-unmask classification model is used to analyze the USA cities Instagram data set. The fit score analysis framework is just used for BLM posts to capture the mask use patterns during a huge social gathering.

**Figure 1:**
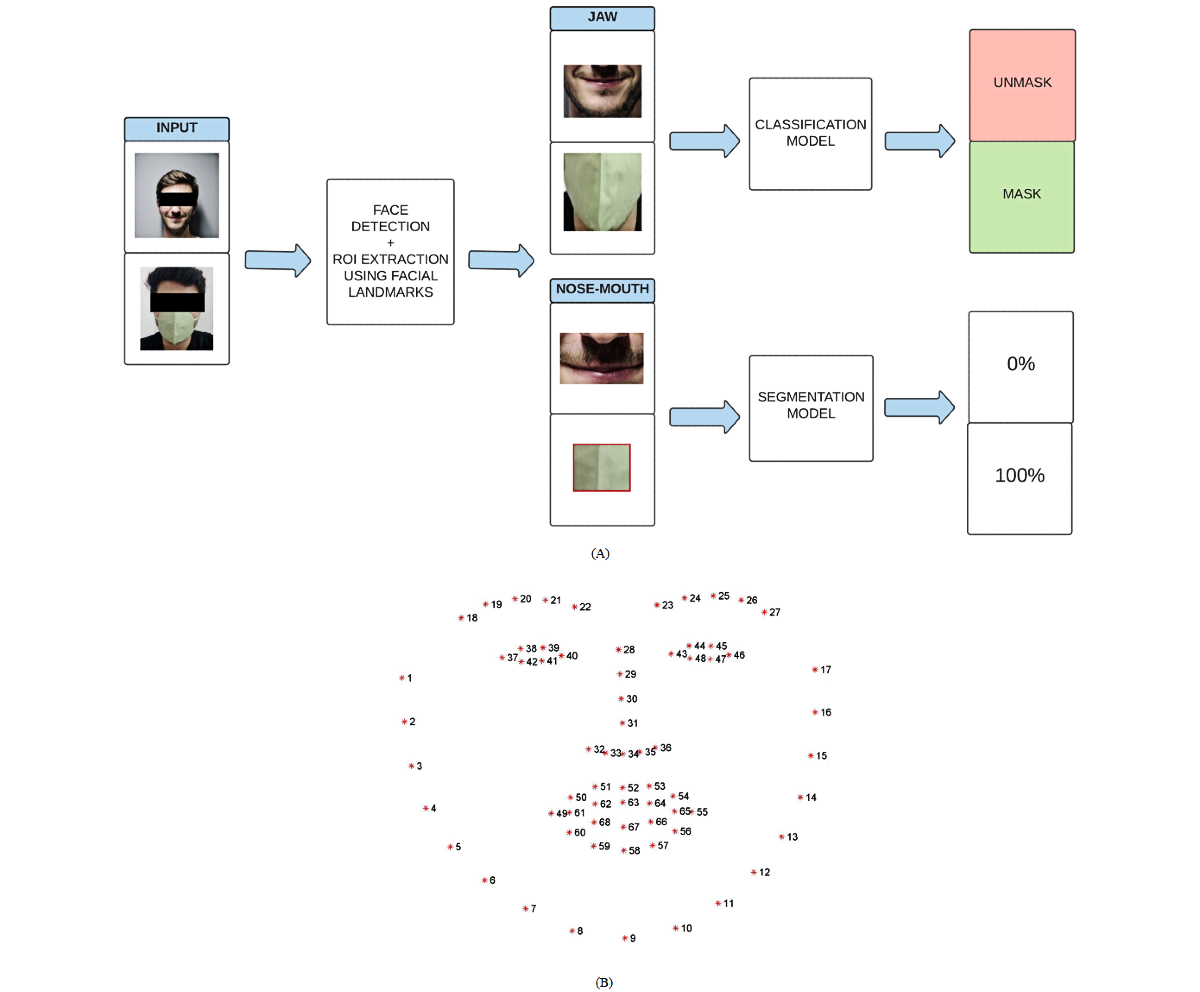
(A) Face mask detection and mask fit calculation framework. The extracted jaws are passed to the trained mask-unmask classification model. The extracted nose-mouth region is given to the segmentation model to predict the masked region and calculate the fit score. (B) Facial landmarks detected on a face using Dlib. ROI: region of interest.

Images obtained from sources mentioned in the previous section consisted of various individuals. Thereby, to detect face masks in these images, the first task of both frameworks was face detection. This was done using the pretrained model Retinaface [33], which is one of the top performing models on Face Detection on the WIDER Face (Hard) data set [34]. Next, facial landmarks were obtained using Dlib’s implementation [35] (proposed by Kazemi et al [36]), which was used to extract the regions of interest (ROIs), as shown in Figure 1A. The landmarks 5-13, 31-36, and 49-68 (Figure 1B) were used to filter the face’s jaw region. This jaw region obtained was then used as input in the classification model for the mask-unmask classification framework. The landmarks 32-36 and 49-68 (Figure 1B) were used to filter the nose-mouth region from the face. This nose-mouth region obtained was then used for calculating the fit score.

The jaw region was then classified on the basis of whether it contained a mask over it or not (Figure 1A) using a classification model. The following architectures were experimented with while training the mask-unmask classification model: MobileNet V2, Nas Net, EffecientNet B0, EffecientNet B1, EffecientNet B2, and DenseNet121. These architectures were selected since they have significantly fewer parameters than most other architectures (Multimedia Appendix 1). The input image size for all the models was 224×224. Transfer learning was used, and weight initialization of all models was done using ImageNet. All models were truncated at the last fully connected layer. The following layers were added: (1) average pooling with 5×5 pool size, (2) flatten layer, (3) dense layer with 128 hidden units and reLU activation, (4) dropout layer of 0.5, and (5) dense layer of 2 hidden units and Softmax activation. Adam optimizer with an initial learning rate of 1e-4 was used, and each model was trained for 30 epochs with a batch size of 64, with binary cross-entropy as the loss function. For training the classifier, the total data from the mask-unmask classifier data set consisted of 9055 masked and 9055 unmasked samples, which were split in an 80:20 ratio for training and validation sets. Five-fold cross-validation was used to evaluate the trained models’ performance, and the different model results can be found in Multimedia Appendix 1.

The evaluation indicated that EfficientNet B0 was the best performing model, with an overall accuracy of 0.98 (SD 0.01). EfficientNet [37] is a convolutional neural network architecture and scaling method that uniformly scales all depth/width/resolution dimensions. Efficient B0 has 5.3 million parameters with 18 layers. Its architecture consists of an initial 3×3 convolution layer, followed by a series of MBconv layers with different kernel sizes and number of channels. The series of MBconv layers are followed by a convolution layer, a pooling layer, and a fully connected layer. It uses linear activation in the last layer in each block to prevent loss of information from ReLU. Compared with conventional convolutional neural network models, the main building block for EfficientNet is MBConv, an inverted bottleneck conv, known initially as MobileNetV2. Before EfficientNet came along, the most common way to scale up ConvNet was by one of the following 3 dimensions: depth (number of layers), width (number of channels), and image resolution (image size). EfficientNet, on the other hand, performs compound scaling, that is, scaling of all 3 dimensions while maintaining a balance between all dimensions of the network. We used this trained model for further analysis using the mask-unmask classification framework.

To calculate the fit score of the appropriate region covering the nose and mouth regions of the face, we used a semantic segmentation-based model (Figure 1A). We defined the fit score as shown in Equation 1. Using the data from the fit score data set, we trained a U-Net–based model [38] for segmenting images of faces into the masked and unmasked regions. The model uses ResNet 32 and 50 encoders pretrained on ImageNet data [39]. The layers were trained progressively using cyclical learning rates [40]. Different model variations were experimented with using different encoders and input image sizes (Multimedia Appendix 2). Using the output of this model (true positive [TP] + false positive [FP]) and the nose-mouth region’s facial landmarks, we calculated the fit score of an image of a face. The fit score is the percentage of ROI area covered by the mask on the face (Figure 1A). We employed the fit score analysis framework on BLM posts to understand how well people wore masks in groups during large events like protests. City-wise analysis was done to observe mask fit differences across major states of protest.




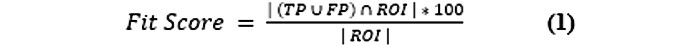




### Statistical Tests

We used the Mann-Kendall trend test to look for monotonic increasing trends in the daily percentage of mask users.

We also used Pearson correlation, Spearman rank-sum correlation, and the Welch *t* test to perform our analysis. Although Pearson correlation assumes normal distribution for both variables [41], it has been shown to reveal hidden correlations even when data are not normally distributed [42].

We performed Pearson and Spearman correlations to decide whether the value of the correlation coefficient *r* between lagged COVID-19 cases and the daily percentage of people wearing masks is significantly different from 0 at a threshold of *P*<.01 [43].

We then conducted the Welch *t* test to assess whether the daily posting is significantly affected by stay-at-home laws. The Welch *t* test requires normal distribution as a prerequisite, but since we were comparing mean values and our underlying series length was large (>30), this assumption could be bypassed [44]. We assessed the before and after posting effects of the application of stay-at-home laws in New York, Dallas, Seattle, Boston, Minneapolis, and New Orleans (Multimedia Appendix 3) [45-50]. We perform the Welch *t* test to test the following hypotheses for the 6 cities and calculate the *P* values with an alpha of .01: H0, μ0=μ1 (the daily percentage of group posting is not affected by the stay-at-home laws) and H1, μ0≠μ1 (the daily percentage of group posting is affected by the stay-at-home laws), where μ0 and μ1 are the mean percentages of daily group posting.

In addition, we performed the Welch *t* test to test the effect on the percentage of masked faces from mask mandates for Boston, Minneapolis, and New York City (mask mandate dates for the other 3 cities did not lie in our chosen timeframe) (Multimedia Appendix 4) [45,51,52]. The hypotheses are as follows: H0, μ0=μ1 (the daily percentage of masked faces is not affected by the mask mandates) and H1, μ0≠μ1 (the daily percentage of masked faces is affected by the mask mandates), where μ0 and μ1 are the daily mean percentages of masked faces. We calculated the associated *P* value for significance testing, with an alpha of .01.

### Interpretability

**Figure 2:**
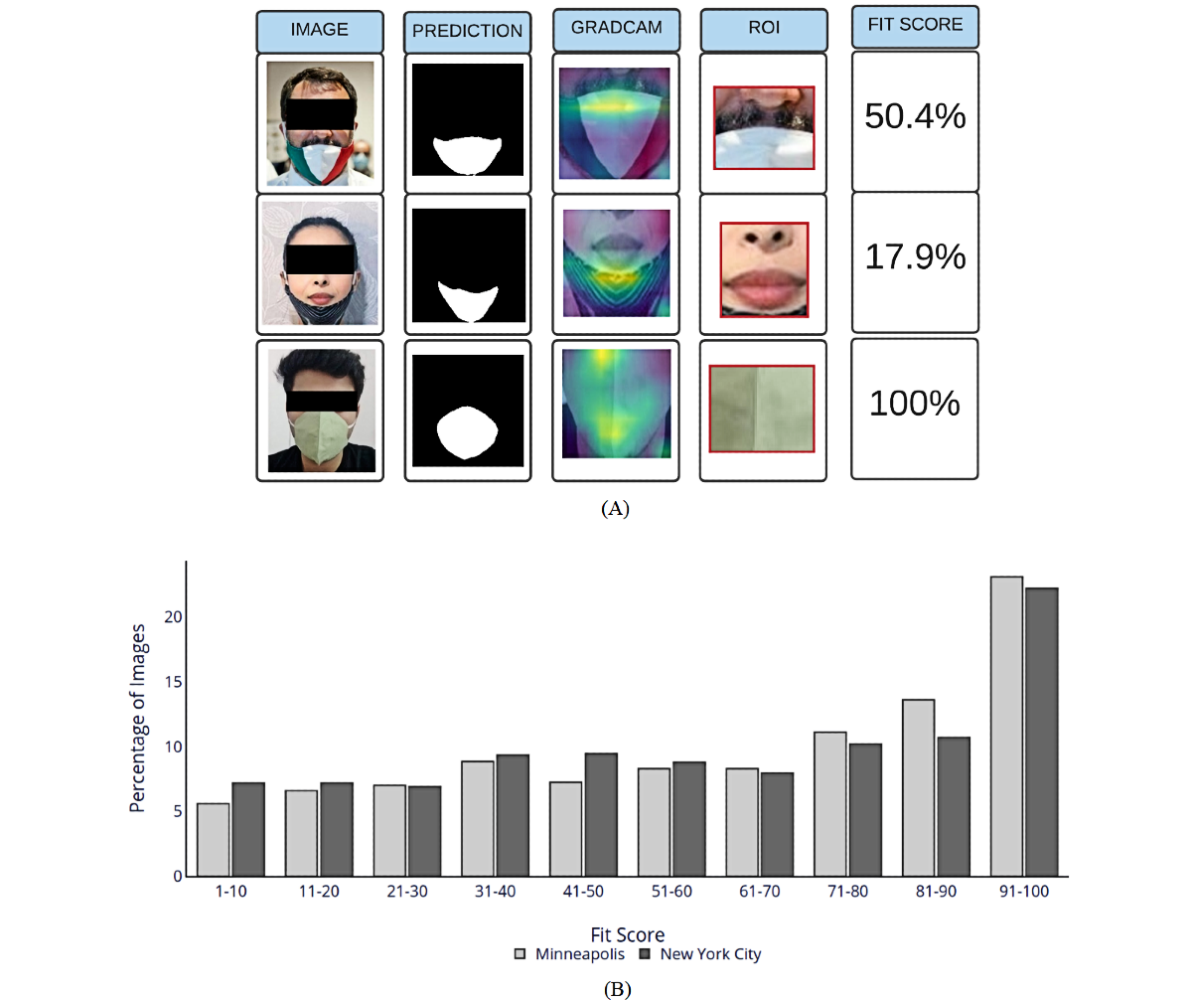
(A) GradCam analysis showing the activation of different regions on the jaw in the classification model. (B) Percentage of faces vs fit score for New York and Minneapolis for Black Lives Matter posts between May 25, 2020, and July 15, 2020. A total of 11,214 posts were analyzed. ROI: region of interest.

To visually inspect what the trained EfficientNet B0 in the mask-unmask classifier had learned, we implemented GradCam [53] on the network. GradCam assesses which parts of the input image have the highest activation values, given a target class. In this case, we passed the jaw region (ROI) after facial landmark detection as the input image to the GradCam network for 3 examples (2 masked and 1 unmasked) (Figure 2A). We also inspected the segmentation model on the corpus of BLM posts collected from social media.

## Results

The corresponding activation maps, mask predictions, and fit scores from GradCam analysis are shown in Figure 2A. Figure 2B shows the distribution of the fit scores for people wearing masks in BLM posts. Approximately 35% of the detected faces had a fit score ≥80% (the corresponding n/N values can be found in Multimedia Appendix 5). This means that the remaining 65% had some significant part of their nose/mouth region not covered.

The following paragraph presents the results of experiments conducted to evaluate the patterns of people wearing masks in the 6 cities across the selected time frame (February 1, 2020, and May 31, 2020). A total of 1.66 million faces were detected from all the posts across the 6 cities. Out of which, a total of 232,706 faces had masks. Table 1 shows the city-wise distribution of the detected faces and masks. We found that 1.16 million posts (around 57% of the total posts collected) had no faces, while 1.89 million (around 93%) of the total posts had no masked faces. One or more faces were detected in 0.87 million (43%) of the posts, of which 0.61 million (30%) had a single face detected and 0.26 million (13%) had multiple faces detected. In 0.14 million (7%) of the total posts, one or more masked faces were detected, out of which 0.12 million (6%) had a single masked face.

**Figure 3:**
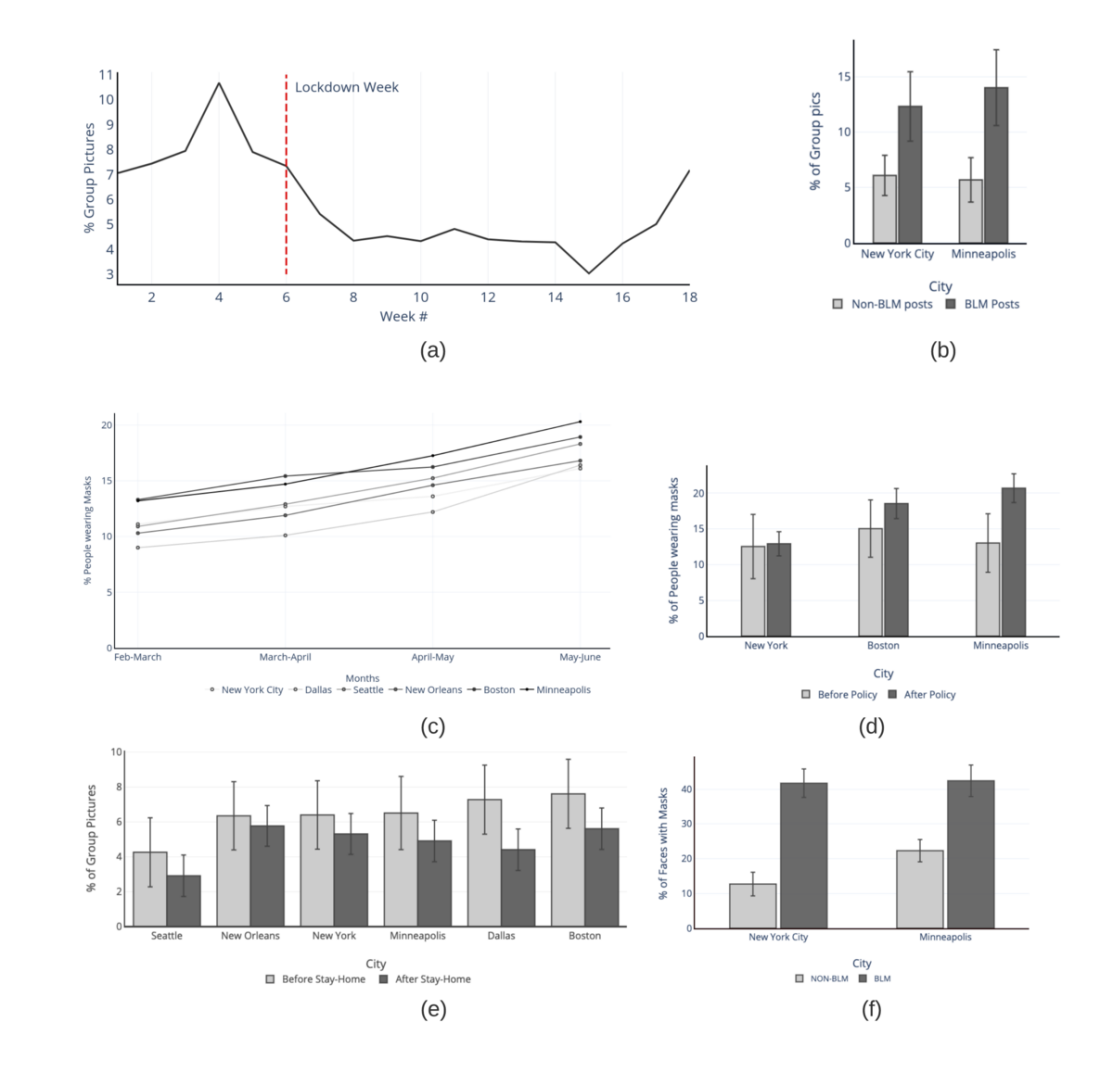
(A) Weekly percentages of group pictures detected from New York City, Seattle, Dallas, New Orleans, Minneapolis, and Boston between February 1, 2020, and May 31, 2020. A total of 2.04 million posts were analyzed. (B) Percentage of group pictures vs city for Black Lives Matter (BLM) and non-BLM posts between May 25, 2020, and July 15, 2020. A total of 192,854 posts were analyzed. (C) Monthly percentages of people wearing masks for each of the 6 cities, between February 1, 2020, and May 31, 2020. The data set was divided into months for each city, and the percentages of people wearing masks were computed. (D) Average daily percentage of people wearing masks before and after mask use guidelines for New York, Boston, and Minneapolis, between February 1, 2020, and May 31, 2020. A total of 750,433 posts were analyzed. (E) Average daily percentage of group pictures before and after stay-at-home laws for the 6 cities between February 1, 2020, and May 31, 2020. A total of 2.04 million posts were analyzed. (F) Percentage of people wearing masks in groups for BLM and non-BLM posts between May 25, 2020, and July 15, 2020. A total of 27,789 posts were analyzed.

There was a decrease in group posting after the lockdown week. The average percentage of group pictures dropped from 8.05% to 4.65%. A sudden spike in group posting was observed around week 15 (Figure 3A).

A general increasing trend in the percentage of people wearing masks for all 6 cities was observed. The Mann-Kendall trend test showed a significant positive trend in the daily percentage of mask users for all 6 cities (corresponding *P* values can be found in Multimedia Appendix 6). New York City, Dallas, Seattle, New Orleans, Boston, and Minneapolis observed a month-wise increase of 5%, 7.4%, 7.4%, 6.5%, 5.6%, and 7.1%, respectively, between February 2020 and May 2020 (Figure 3C) (the corresponding n/N values can be found in Multimedia Appendix 7).

As shown in Figure 3B, the differences in group pictures between BLM and non-BLM posts were 6.2% and 8.3% for New York City and Minneapolis, respectively. The differences in the percentage of masked faces in group pictures between BLM and non-BLM posts were 29.0% and 20.1% for New York City and Minneapolis, respectively (Figure 3F).

Figure 3D shows the average daily percentage of people wearing masks before and after the state mask mandates were applied for the 3 cities that implemented these mandates within our selected time range. Boston, Minneapolis, and New York City saw increases of 3.0%, 7.4%, and 1.0%, respectively, after applying mask mandates (the corresponding n/N values can be found in Multimedia Appendix 8). The average daily percentages of people wearing masks before and after the state mask mandates were applied were statistically different from one another for Boston and Minneapolis, with an alpha of .01, while the difference was not significant for New York City (test results can be found in Multimedia Appendix 9).

Boston, Minneapolis, New Orleans, Dallas, Seattle, and New York City saw decreases of 2.0%,1.6%, 0.6%, 2.8%, 1.3%, and 1.0%, respectively, in the average daily percentage of group pictures before and after the stay-at-home laws (the corresponding n/N values can be found in Multimedia Appendix 10) (Figure 3E). The average daily percentages of group posting before and after the stay-at-home laws were applied were statistically different from one another for all the 6 cities, with the alpha value set at .01 (test results can be found in Multimedia Appendix 11).

**Table 1:** City-wise distribution of the number of detected faces, number of detected masks, and number of masks per face through our framework, collected from Instagram between February 1, 2020, and May 31, 2020.

City	Total collected posts, n	Faces detected, n	Masks detected, n	Percentage of faces with masks
New York City	245,677	200,089	25,413	12.70
Dallas	540,500	444,194	48,119	10.83
Seattle	437,040	312,012	46,019	14.75
Minneapolis	220,999	152,822	30,385	19.88
New Orleans	315,082	321,591	39,420	12.26
Boston	283,757	238,770	43,350	18.15
Total	2,043,055	1,669,478	232,706	13.94

## Discussion

### Principal Findings

The COVID-19 pandemic has given the entire research community and governments a chance to reflect on what kind of system needs to be in place to handle such catastrophes. A renewed focus is emerging in infodemiology [54], especially leveraging mass surveillance data [55,56]. Location tracking [57], periodic self-checks, and image recognition systems have been deployed by many governments [58-61] to get a handle on the pulse of the pandemic in their states. This study suggests another such approach, which can be applied to specific demographics to achieve similar near–real-time tracking of the pandemic’s spread. Instagram and other social media platforms have been very successful in tracking the number of visits to public places [62]. In the context of COVID-19, public places are the focal points for the spread of the virus. It has been well-documented that face masks and social distancing are the 2 most effective nonpharmaceutical interventions to curb the spread of COVID-19 [7]. However, the use of masks and effective social distancing are often self-reported [63], without any proof to corroborate the claims. Models built on image data with location data [64] can be a powerful tool for the authorities to keep track of the pandemic’s pulse.

**Figure 4:**
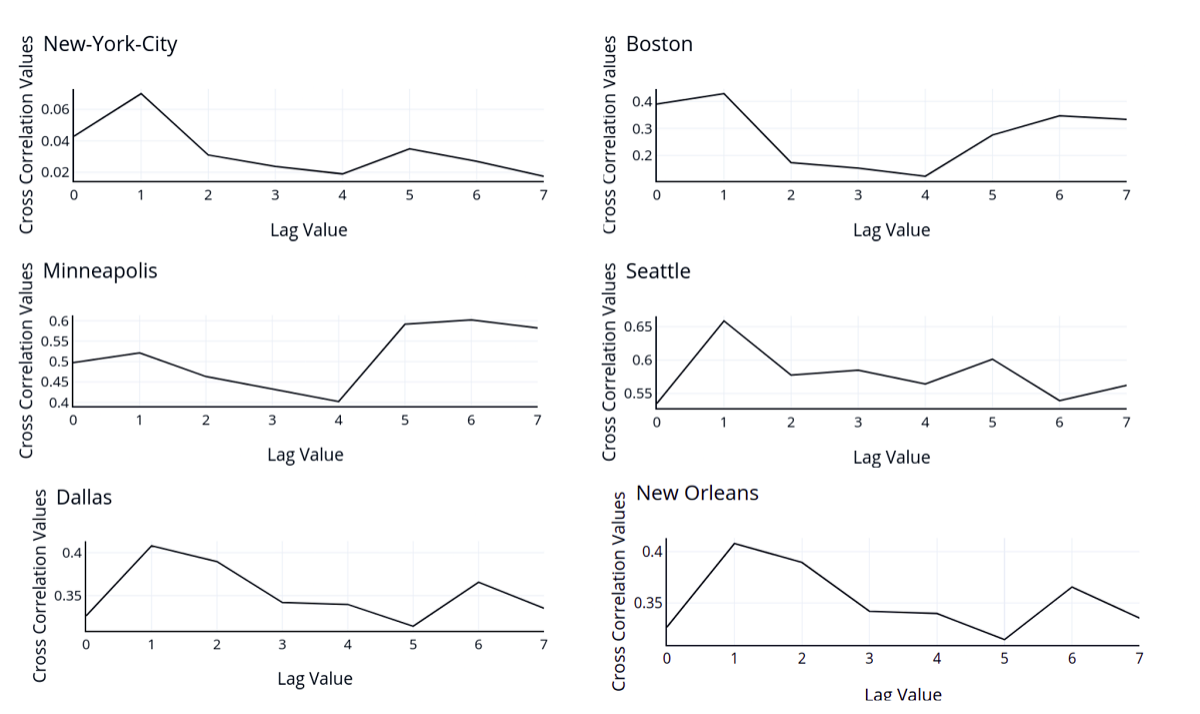
Pearson correlation between daily lagged cumulative cases and the percentage of masked photos between February 1, 2020 and May 31, 2020. The length of the series was 120. The lag was selected between 0 and 7 based on the highest correlation value.

An overall decrease in group posting was found as the pandemic grew and lockdowns were put in place. These group pictures posted online can be used as an estimator for the percentage of people spending time in groups. A sudden spike in group posting was found from May 16, 2020, to May 22, 2020 (week 15 of our timeline). This can be linked to the easing lockdown restrictions through weeks 13, 14, and 15 [65-68]. As the pandemic spread, the percentage of mask users saw a significant increasing trend for all the 6 cities through the months, suggesting that more people started wearing masks as the pandemic spread. Significant positive Spearman and Pearson correlations were found between the daily COVID-19 cases and the percentage of mask users for all the cities, except New York City, with an alpha of .01 (Multimedia Appendices 12 and 13). The maximum correlation was found to be with lag as 1 for all cities, except Minneapolis, as seen in Figure 4. As seen through social media, the stay-at-home state policies were successful as a significant decrease in group posting was observed on the adoption of stay-at-home laws for all 6 analyzed cities. After the mask use mandates were applied, a significant increase in the percentage of mask users was seen in Boston and Minneapolis, and a slight increase was observed in New York City. These results indicate adherence to nonpharmaceutical interventions in the 6 cities, with varying percentages of changes and effects. The trends of increasing mask use with the pandemic and positive changes with mask mandates corroborate with self-reported number-based survey methods in the United States [7]. Although a significant increase was seen in the percentages, the growth could have increased separately from the mandates. With an insignificant increase seen for New York City, supplemental public health interventions can be applied to maximize the adoption of such methods.

A large difference was observed in the percentage of group pictures between the posts that talked about the BLM protests. This difference can be explained by the huge collection of people in protests, with a lack of social distancing measures causing a high percentage of posts to involve group pictures. On the contrary, mask use was found to be much higher for BLM-related posts as compared to non-BLM posts. The mask fit score distribution of the protestors showed that only 35% of mask users had more than 80% of their nose/mouth region covered. This indicates that, while social distancing measures were not appropriately followed due to the nature of such large gatherings, protestors were more likely to wear a mask than the general public, but only a small percentage covered their faces properly, as seen through social media posts.

Models built on image data with location data can be powerful tools for authorities to keep track of the pandemic’s pulse. This study provides a new method for governments and organizations to monitor policy decisions indirectly.

### Limitations

The images present in our data sets have a high definition and are in RGB mode. If the trained models have to be deployed in a new setting (eg, CCTV feed), certain image augmentation techniques like gray scaling and rescaling might be needed to fine-tune the models. For the analysis, we chose the image data from 6 major US cities by population and correlated the data with state-wide COVID-19 cases. Since these cities are some of the most populated cities of their respective states, it is reasonable to assume that they will be the hotbeds of COVID-19 spread in their respective states.

The analysis was conducted using images obtained from the social media platform Instagram. We understand that these images might not represent the entire city population [69]. However, they do represent a wide demographic of internet users [70]. With recent studies conducted on geo-tagged text data present in Instagram posts [71], our assumption of a fair population representation in the Instagram data might not be too far-fetched. Among the mask users, celebrity posts (posts with likes greater than 10,000) contributed to only 0.2% of the total posts, which contained at least one mask, showing that the collected data mainly involved posts from the general population. However, we do acknowledge that capturing metadata for users while performing similar studies might yield a conclusive answer to the question of fair representation. Capturing and using metadata can be future work, which will build on our results.

### Future Work

An addition to the modeling pipeline could be an indoor/outdoor environment detector, similar to that in the study by Zhou et al [72]. Another addition to the analysis could be selectively looking at the specific activity of users, who are deemed as “influencers” on the network. Their activity on the network can be analyzed in conjunction with the activity of their followers. This can help determine the role of social networks and the power of certain influential nodes in that network over other people’s behavior during critical times such as a pandemic. Dynamic location relationships present in mobility data [73] can be further used to understand the pandemic’s spread with higher location precision and even recognize malevolent actors in the system. A natural extension of this work is its replication across different social media platforms like Twitter, Facebook, and Baidu.

### Conclusions

Models built on image data with location data can be powerful tools for authorities to keep track of the pandemic’s pulse. This study examined 2.04 million posts collected from 6 US cities between February 1, 2020, and May 31, 2020, for adherence to mask use and social distancing, as seen through social media.

This study found a general increasing trend in mask use and a decreasing trend in group pictures as the pandemic spread. The stay-at-home laws caused a significant drop in group posting for all 6 cities, while the mask mandates caused a significant increase in mask use for 2 of the 3 cities analyzed. Although these results suggest an upward trend in the adoption of preventive methods, a large portion of nonadopters seen online indicates a need for supplemental measures to increase the effectiveness of such methods.

Posts related to protests were found to capture the lack of attention given to safety measures, with high percentages of detected group pictures and incorrect mask use. The methodology used provides a directional indication of how government policies can be indirectly monitored. The findings can help governments and other organizations as indicators for the successful implementation of nonpharmaceutical interventions for the COVID-19 pandemic.

## Appendix

Multimedia Appendix 1 Face mask detection model results.

Multimedia Appendix 2 Face mask fit analyzer model results.

Multimedia Appendix 3 Dates on which stay-at-home guidelines were enacted by the respective state governments.

Multimedia Appendix 4 Dates on which mask mandates were enacted by the respective state governments.

Multimedia Appendix 5 Underlying n and N values for Figure 2B.

Multimedia Appendix 6 Test statistics and *P* values for the Mann-Kendall trend test for the daily percentage of mask wearers in 6 cities.

Multimedia Appendix 7 Underlying n and N values for Figure 3C.

Multimedia Appendix 8 Underlying n and N values for Figure 3D.

Multimedia Appendix 9 Welch *t* test statistics and *P* values to test for equal means before and after the application of mask mandates.

Multimedia Appendix 10 Underlying n and N values for Figure 3E.

Multimedia Appendix 11 Welch *t* test statistics and *P* values to test for equal means before and after the application of stay-at-home orders.

Multimedia Appendix 12 Pearson correlation coefficients and *P* values between lagged cumulative COVID-19 cases and the daily percentage of people wearing masks.

Multimedia Appendix 13 Spearman correlation coefficients and *P* values between lagged cumulative COVID-19 cases and the daily percentage of people wearing masks.

## Abbreviations

BLM: Black Lives Matter
MAFA: Masked Face
RMFD: Real-world Masked Face Dataset
ROI: region of interest
