# Supplementary material for: COVID-19 Mask Usage and Social Distancing in Social Media Images: Large-scale Deep Learning Analysis
Source: JMIR Public Health Surveill. 2022 Jan 18;8(1):e26868. doi: 10.2196/26868 (PMC8768939; doi:10.2196/26868)
Supplement: Multimedia Appendix 1 [file publichealth_v8i1e26868_app1.docx]

**Multimedia Appendix 1.** Face mask detection model results.

| Model | No. of Parameters | Recall | Accuracy |
| --- | --- | --- | --- |
|  |  |  |  |
| MobileNet V2 | 3,538,984 | 0.90 ± 0.01 | 0.94 ± 0.01 |
| Nas Net Mobile | 5,326,716 | 0.86 ± 0.01 | 0.91 ± 0.01 |
| EffecientNet B0 | 5,330,571 | 0.97 ± 0.01 | 0.98 ± 0.00 |
| EffecientNet B1 | 7,856,239 | 0.97 ± 0.01 | 0.98 ± 0.00 |
| DenseNet121 | 8,062,504 | 0.90 ± 0.01 | 0.94 ± 0.01 |
| EffecientNet B2 | 9,177,569 | 0.97 ± 0.01 | 0.98 ± 0.00 |

EffecientNet B0 is used as the model for face mask detection due to its superior performance and smaller model size. The precision for each model was found to be 1.00 ± 0.01
