# Supplementary material for: COVID-19 Mask Usage and Social Distancing in Social Media Images: Large-scale Deep Learning Analysis
Source: JMIR Public Health Surveill. 2022 Jan 18;8(1):e26868. doi: 10.2196/26868 (PMC8768939; doi:10.2196/26868)
Supplement: Multimedia Appendix 2 [file publichealth_v8i1e26868_app2.docx]

**Multimedia Appendix 2.** Face mask fit analyzer model results.

| Image Size | Encoder | Recall | Accuracy | IOU |
| --- | --- | --- | --- | --- |
|  |  |  |  |  |
| 100 x 100 | Resnet 50 | 0.96 | 0.98 | 0.95 |
| 150 x 150 | Resnet 50 | 0.96 | 0.98 | 0.95 |
| 224 x 224 | Resnet 32 | 0.96 | 0.98 | 0.95 |

IOU refers to the Intersection Over Union (IOU) score .

$IOU = \frac{| TP |}{| (TP \cup FN \cup FP) |}$ … (2)

Intersection Over Union (IOU) score is a standard metric used for evaluating semantic segmentation tasks. It represents the fraction of the true positive pixels present in the sum of true and predicted positive pixels.
