# Supplementary material for: COVID-19 Mask Usage and Social Distancing in Social Media Images: Large-scale Deep Learning Analysis
Source: JMIR Public Health Surveill. 2022 Jan 18;8(1):e26868. doi: 10.2196/26868 (PMC8768939; doi:10.2196/26868)
Supplement: Multimedia Appendix 3 [file publichealth_v8i1e26868_app3.docx]

**Multimedia Appendix 3.** Dates on which stay-at-home guidelines were enacted by the respective state governments.

| City | Date | Comment |
| --- | --- | --- |
|  |  |  |
| Boston | March 23,2020 | Boston Department of Public Health issued a two-week stay-at-home advisory on March 23, 2020 [2] |
| Minneapolis | March 27, 2020 | For Minneapolis, stay-at-home advisory came into effect on March 27, 2020 [3] |
| New Orleans | March 20, 2020 | New Orleans enacted stay-at-home orders starting March 20, 2020 [4] |
| Dallas | March 23, 2020 | Dallas enacted stay-at-home orders starting March 23, 2020 [26]. |
| Seattle | March 23, 2020 | Seattle enacted stay-at-home orders starting May 31, 2020 [5]. |
| New York | March 20, 2020 | In New York State state-wide stay-at-home order was declared on March 20, 2020 [6] |
