# Supplementary material for: COVID-19 Mask Usage and Social Distancing in Social Media Images: Large-scale Deep Learning Analysis
Source: JMIR Public Health Surveill. 2022 Jan 18;8(1):e26868. doi: 10.2196/26868 (PMC8768939; doi:10.2196/26868)
Supplement: Multimedia Appendix 4 [file publichealth_v8i1e26868_app4.docx]

**Multimedia Appendix 4.** Dates on which mask mandates were enacted by the respective state governments.

| City | Date | Comment |
| --- | --- | --- |
|  |  |  |
| Boston | May 6, 2020 | In Boston, residents were asked to wear a mask in public places starting May 6, 2020 [2] |
| Minneapolis | April 30, 2020 | Mask wearing guidelines were put in place on April 30, 2020 for Minneapolis, encouraging people to wear face coverings [7] |
| New York State | April 15, 2020 | In New York State, residents were asked to wear masks in public starting April 15, 2020 [8] |
