# Supplementary material for: COVID-19 Mask Usage and Social Distancing in Social Media Images: Large-scale Deep Learning Analysis
Source: JMIR Public Health Surveill. 2022 Jan 18;8(1):e26868. doi: 10.2196/26868 (PMC8768939; doi:10.2196/26868)
Supplement: Multimedia Appendix 5 [file publichealth_v8i1e26868_app5.docx]

**Multimedia Appendix 5.** Underlying n and N values for Figure 2B.

| Fit Score | Minneapolis | New York City |
| --- | --- | --- |
|  |  |  |
| 1-10 | 1281/23805 | 1659/24038 |
| 11-20 | 1576/23805 | 1732/24038 |
| 21-30 | 1687/23805 | 1681/24038 |
| 31-40 | 2104/23805 | 2254/24038 |
| 41-50 | 1747/23805 | 2316/24038 |
| 51-60 | 1981/23805 | 2130/24038 |
| 61-70 | 1995/23805 | 1939/24038 |
| 71-80 | 2632/23805 | 2466/24038 |
| 81-90 | 3233/23805 | 2559/24038 |
| 91-100 | 5569/23805 | 5302/24038 |
