# Supplementary material for: COVID-19 Mask Usage and Social Distancing in Social Media Images: Large-scale Deep Learning Analysis
Source: JMIR Public Health Surveill. 2022 Jan 18;8(1):e26868. doi: 10.2196/26868 (PMC8768939; doi:10.2196/26868)
Supplement: Multimedia Appendix 6 [file publichealth_v8i1e26868_app6.docx]

**Multimedia Appendix 6.** Test statistics and *P* values for the Mann-Kendall trend test for the daily percentage of mask wearers in 6 cities.

| City | Normalized test statistic (z) | *P Values* |
| --- | --- | --- |
|  |  |  |
| New York City | 4.1 | <.001 |
| Boston | 6.34 | <.001 |
| Minneapolis | 5.65 | <.001 |
| Seattle | 8.72 | <.001 |
| Dallas | 8.22 | <.001 |
| New Orleans | 8.47 | <.001 |
