# Supplementary material for: COVID-19 Mask Usage and Social Distancing in Social Media Images: Large-scale Deep Learning Analysis
Source: JMIR Public Health Surveill. 2022 Jan 18;8(1):e26868. doi: 10.2196/26868 (PMC8768939; doi:10.2196/26868)
Supplement: Multimedia Appendix 7 [file publichealth_v8i1e26868_app7.docx]

**Multimedia Appendix 7.** Underlying n and N values for Figure 3C.

| Time Period | New York City | Dallas | Seattle | New Orleans | Boston | Minneapolis |
| --- | --- | --- | --- | --- | --- | --- |
|  |  |  |  |  |  |  |
| Feb-March | 4847/45301 | 10369/112882 | 6211/56650 | 13537/131390 | 9863/73506 | 5373/36741 |
| March-April | 5983/49869 | 11438/113014 | 9105/70312 | 9305/80011 | 13398/86987 | 6734/42279 |
| April-May | 7545/58310 | 24878/209186 | 9958/65373 | 7083/49809 | 600/3649 | 2869/16139 |
| May-June | 7038/46609 | 1438/9112 | 20745/119677 | 9545/60381 | 16280/75678 | 12183/49777 |
