# Supplementary material for: COVID-19 Mask Usage and Social Distancing in Social Media Images: Large-scale Deep Learning Analysis
Source: JMIR Public Health Surveill. 2022 Jan 18;8(1):e26868. doi: 10.2196/26868 (PMC8768939; doi:10.2196/26868)
Supplement: Multimedia Appendix 8 [file publichealth_v8i1e26868_app8.docx]

**Multimedia Appendix 8.** Underlying n and N values for Figure 3D.

| City | Sum of daily percentages/total days before mandate | std Dev | Sum of daily percentages/total days after mandate | std Dev |
| --- | --- | --- | --- | --- |
|  |  |  |  |  |
| New York State | 927.96/74 | 4.5 | 594.32/46 | 1.7 |
| Boston | 1428.8/95 | 4.01 | 463.75/25 | 2.1 |
| Minneapolis | 1159.67/89 | 4.1 | 952.2/46 | 2.01 |
