# Supplementary material for: COVID-19 Mask Usage and Social Distancing in Social Media Images: Large-scale Deep Learning Analysis
Source: JMIR Public Health Surveill. 2022 Jan 18;8(1):e26868. doi: 10.2196/26868 (PMC8768939; doi:10.2196/26868)
Supplement: Multimedia Appendix 9 [file publichealth_v8i1e26868_app9.docx]

**Multimedia Appendix 9.** Welch *t* test statistics and *P* values to test for equal means before and after the application of mask mandates.

| City | *t* statistic | *P* Values |
| --- | --- | --- |
|  |  |  |
| New York City | 1.68 | .09 |
| Boston | 4.19 | <.001 |
| Minneapolis | 3.64 | <.001 |
