# Supplementary material for: COVID-19 Mask Usage and Social Distancing in Social Media Images: Large-scale Deep Learning Analysis
Source: JMIR Public Health Surveill. 2022 Jan 18;8(1):e26868. doi: 10.2196/26868 (PMC8768939; doi:10.2196/26868)
Supplement: Multimedia Appendix 10 [file publichealth_v8i1e26868_app10.docx]

**Multimedia Appendix 10.** Underlying n and N values for Figure 3E.

| City | Sum of daily percentages/total days before mandate | Std Dev | Sum of daily percentages/total days after mandate | Std Dev |
| --- | --- | --- | --- | --- |
|  |  |  |  |  |
| Seattle | 216.5/51 | 1.98 | 200.1/69 | 1.19 |
| New Orleans | 304.32/48 | 1.96 | 414.72/72 | 1.17 |
| New York | 306.72/48 | 1.96 | 381.6/72 | 1.17 |
| Minneapolis- | 351.45/55 | 2.1 | 344.5/65 | 1.19 |
| Dallas | 370.77/51 | 1.98 | 303.6/69 | 1.19 |
| Boston- | 364.8/48 | 1.98 | 403.2/72 | 1.19 |
