# Supplementary material for: COVID-19 Mask Usage and Social Distancing in Social Media Images: Large-scale Deep Learning Analysis
Source: JMIR Public Health Surveill. 2022 Jan 18;8(1):e26868. doi: 10.2196/26868 (PMC8768939; doi:10.2196/26868)
Supplement: Multimedia Appendix 11 [file publichealth_v8i1e26868_app11.docx]

**Multimedia Appendix 11.** Welch t test statistic and P values to test for equal means before and after the application of stay-at-home orders.

| City | *t* statistic | *P* Values |
| --- | --- | --- |
|  |  |  |
| New York City | -4.35 | <.001 |
| Boston | -6.0 | <.001 |
| Minneapolis | -2.75 | .008 |
| Seattle | -6.53 | <.001 |
| Dallas | -8.86 | <.001 |
| New Orleans | -8.74 | <.001 |
