# Supplementary material for: COVID-19 Mask Usage and Social Distancing in Social Media Images: Large-scale Deep Learning Analysis
Source: JMIR Public Health Surveill. 2022 Jan 18;8(1):e26868. doi: 10.2196/26868 (PMC8768939; doi:10.2196/26868)
Supplement: Multimedia Appendix 12 [file publichealth_v8i1e26868_app12.docx]

**Multimedia Appendix 12.** Pearson correlation coefficients and *P* values between lagged cumulative COVID-19 cases and the daily percentage of people wearing masks.

| City | Max Lag | Correlation Value | *P* Values |
| --- | --- | --- | --- |
|  |  |  |  |
| New York City | 1 | 0.07 | .50 |
| Boston | 1 | 0.42 | <.001 |
| Minneapolis | 6 | 0.60 | <.001 |
| Seattle | 1 | 0.65 | <.001 |
| Dallas | 1 | 0.40 | <.001 |
| New Orleans | 1 | 0.51 | <.001 |
